# Aiding early clinical drug development by elucidation of the relationship between tumor growth inhibition and survival in relapsed/refractory multiple myeloma patients

**DOI:** 10.1002/jha2.494

**Published:** 2022-06-02

**Authors:** Yiming Cheng, Kevin Hong, Nianhang Chen, Xin Yu, Teresa Peluso, Simon Zhou, Yan Li

**Affiliations:** ^1^ Clinical Pharmacology & Pharmacometrics Bristol Myers Squibb New Jersey USA; ^2^ Global Drug Development Bristol Myers Squibb New Jersey USA; ^3^ Global Biometric Sciences Bristol Myers Squibb New Jersey USA; ^4^ Global Drug Development Bristol Myers Squibb Boudry Switzerland

**Keywords:** M‐protein, multiple myeloma, progression‐free survival (PFS), tumor growth inhibition (TGI)

## Abstract

Early prognosis of clinical efficacy is an urgent need for oncology drug development. Herein, we systemically examined the quantitative approach of tumor growth inhibition (TGI) and survival modeling in the space of relapsed and refractory multiple myeloma (MM), aiming to provide insights into clinical drug development. Longitudinal serum M‐protein and progression‐free survival (PFS) data from three phase III studies (*N* = 1367) across six treatment regimens and different patient populations were leveraged. The TGI model successfully described the longitudinal M‐protein data in patients with MM. The tumor inhibition and growth parameters were found to vary as per each study, likely due to the patient population and treatment regimen difference. Based on a parametric time‐to‐event model for PFS, M‐protein reduction at week 4 was identified as a significant prognostic factor for PFS across the three studies. Other factors, including Eastern Cooperative Oncology Group performance status, prior anti‐myeloma therapeutics, and baseline serum ß2‐microglobulin level, were correlated with PFS as well. In conclusion, patient disease characteristics (i.e., baseline tumor burden and treatment lines) were important determinants of tumor inhibition and PFS in MM patients. M‐protein change at week 4 was an early prognostic biomarker for PFS.

## 1 INTRODUCTION

One of the major challenges for oncology drug development is to tease out markers at the early stage to support the prognosis of clinical outcomes of a new treatment option. Depending on disease settings, response assessments, including the Response Evaluation Criteria in Solid Tumors (RECIST) and International Myeloma Working Group (IMWG) [1, 2], have been proposed and widely used in the medical community. The underlying rationale was that response categories are strongly correlated with survival.

However, advances in quantitative methodologies have pivoted the understanding of how to leverage holistic data at an early stage to better interpret the ultimate clinical benefit of the treatment regimen. Tumor growth inhibition (TGI) modeling, which links tumor kinetics to long‐term survival (e.g., progression‐free survival [PFS] and overall survival [OS]), has shown great potential to inform drug development across a wide range of modalities [3, 4, 5, 6, 7, 8, 9, 10]. One of the advantages of such analysis was that rather than diluting the available longitudinal data points into a single outcome measurement of response (e.g., partial response), it utilized all available tumor kinetic data for efficacy assessment [7, 11].

Although the value of TGI has been adequately manifested in the volume of solid tumor indications [8], its application is still rare in hematological malignancies, partly due to the unavailability of actual tumor size measurements and the challenge of finding a reliable tumor burden surrogate. Despite all these above, recent publications by Jonsson et al. [11], Dimopoulos et al. [12], and Bruno et al. [13] have examined the application of TGI in multiple myeloma (MM) by using monoclonal protein (M‐protein) as a surrogate. Biologically, M‐protein is an abnormal immunoglobulin fragment or immunoglobulin light chain secreted by malignant plasma cells. Clinically, M‐protein is a hallmark for MM and constitutes the criteria for IMWG response assessment [1]. Therefore, the use of M‐protein to prognose clinical benefits in the space of MM was evidently justifiable. Additionally, the above publications, although they differed in treatment regimens, unanimously revealed that early M‐protein reduction is highly correlated with long‐term survival, which was in line with other findings from solid tumor indications [3].

The increasing medical needs for patients bearing hematological malignancies and the yet incurable cancers (i.e., MM) have requested drug development to be more than ever prominent. Herein, we described the application of TGI in the space of MM by using data from three phase III clinical studies based on two approved drugs, pomalidomide (Pom) and lenalidomide (Len). Pom and Len were immunomodulatory agents with similar mechanisms of action [14]. Pom, in combination with dexamethasone (dex), was indicated for patients with MM who had received at least two prior therapies, including Len and a proteasome inhibitor and have demonstrated disease progression on or within 60 days of completion of the last therapy [15]. Len in combination with dex was indicated for the treatment of adult patients with MM [16]. The goal of this integrated meta‐analysis is to provide insights into MM drug development, including triaging lead compounds, rationalizing early clinical study design, and informing dosing decisions.

## 2 METHOD

### 2.1 Studies description

The CC‐4047‐MM‐003 (NCT01311687) study was a phase III, multicenter, randomized, open‐label study to compare the efficacy and safety of Pom in combination with low‐dose dexamethasone (LD‐dex) versus high‐dose dexamethasone (HD‐dex) in subjects with relapsed and refractory MM. A total of 455 subjects were enrolled in the study, with 302 patients in the Pom + LD‐dex arm and 153 patients in the HD‐dex arm. Subjects randomized to the Pom + LD‐dex arm received Pom (4 mg/day on days 1–21 of each 28‐day treatment cycle). The starting dose for LD‐dex for subjects who were ≤75 years of age was 40 mg/day on days 1, 8, 15, and 22 of a 28‐day cycle. The starting dose for LD‐dex for subjects who were >75 years of age was 20 mg/day on days 1, 8, 15, and 22 of a 28‐day cycle. In the HD‐dex arm, the starting dose for subjects who were ≤75 years of age was 40 mg/day on days 1–4, 9–12, and 17–20 of each 28‐day cycle. The starting dose for subjects who were >75 years of age was 20 mg/day on days 1–4, 9–12, and 17–20 of each 28‐day cycle.

The CC‐4047‐MM‐007 (NCT01734928) study was a phase III, multicenter, randomized, open‐label study to compare the efficacy and safety of Pom, bortezomib (BTZ), and LD‐dex versus BTZ and LD‐dex in subjects with relapsed and refractory MM. A total of 559 subjects were enrolled in the study, with 281 patients in the Pom + BTZ + LD‐dex arm and 278 patients in the BTZ + LD‐dex arm. Subjects in the Pom + BTZ + LD‐dex arm received 4 mg Pom on days 1–14 of each cycle. BTZ was given at 1.3 mg/m^2^ on days 1, 4, 8, and 11 during cycles 1–8 and on days 1 and 8 for cycles 9 onward. LD‐dex was given at 20 mg/day (≤75 years old) or 10 mg/day (>75 years old) on days 1, 2, 4, 5, 8, 9, 11, and 12 during cycles 1–8 and on days 1, 2, 8, and 9 for cycles 9 onward. Subjects in the BTZ + LD‐dex arm received BTZ 1.3 mg/m^2^ on days 1, 4, 8, and 11 during cycles 1–8 and on days 1 and 8 for cycles 9 onward. LD‐dex was given at 20 mg/day (≤75 years old) or 10 mg/day (>75 years old) on days 1, 2, 4, 5, 8, 9, 11, and 12 during cycles 1–8 and on days 1, 2, 8, and 9 for cycles 9 onward.

CC‐5013‐MM‐009 (NCT00056160) study was a phase III, multicenter, randomized, parallel‐group, double‐blinded, placebo‐controlled study of Len + dex versus dex alone in previously treated subjects with MM. A total of 353 subjects were included in the study, with 177 subjects in the Len + dex arm and 176 subjects in the dex arm. Subjects in the Len + dex arm took 25 mg of Len orally once daily on days 1–21 and a matching placebo capsule once daily on days 22–28 of each 28‐day cycle. Subjects in the placebo/dex arm took one placebo capsule on days 1–28 of each 28‐day cycle. Subjects in both arms took 40 mg of dex orally once daily on days 1–4, 9–12, and 17–20 of each 28‐day cycle for the first four cycles of therapy. Beginning with cycle 5, the dose of dex was reduced to 40 mg orally once daily on days 1–4 for the remaining cycles.

Across all the studies, measurable M‐proteins were part of the inclusion criteria. The M‐protein quantification method was consistent across the three studies. All serum M‐protein electrophoresis was performed at screening and at every cycle on day 1, at treatment discontinuation, every 21 days during the PFS follow‐up phase, and at PFS follow‐up phase discontinuation, if applicable, across all the three studies.

The studies were conducted in accordance with the ethical principles of Good Clinical Practice. All subjects gave written informed consent prior to enrollment. The studies were approved by the Independent Ethics Committee/Institutional Review Boards of the participating center and were conducted according to the Declaration of Helsinki and the International Conference on Harmonization (ICH) Guidelines for Good Clinical Practice. The full study design and clinical results have been detailed in the respective publications [17, 18, 19].

### 2.2 TGI model

The TGI model was developed to account for the dynamics of tumor growth and tumor shrinkage based on a previous publication [20]. Of note, another parsimonious model [3] was also explored but showed inferior model performance. As such, the exponential tumor growth and shrinkage model was used for the analyses in this article.

The TGI model is described by the following equation (Equation (1)), where *y*(*t*) is the M‐protein concentration at time *t* with the value *y*_0_ at baseline (i.e., the M‐protein level at the cycle 1 day 1 visit; fixed to the observed values), SR is the tumor inhibition rate, which differed according to treatment regimens, and PR is the tumor growth rate.

(1) $$y\left(t\right)=\;y_{0}\;\left(e^{-\mathrm{SR}\times t}+e^{\mathrm{PR}\times t}-1\right)$$

The TGI model was used to estimate early changes in M‐protein from baseline at specific timepoints, that is, week 4.

(2) $$\mathrm{week}\left(n\right)\_c\;=\;\frac{M-protein\;\mathrm{level}\;\mathrm{at}\;\mathrm{week}\left(n\right)-\mathrm{baseline}}{\mathrm{baseline}}$$

where week(*n*)_c represents the change in M‐protein at week *n* from baseline and *n* is the integer, which denotes the number of weeks after treatment.

Subject‐level log‐normal distributed random effects were allowed on all the model parameters to account for intersubject variability. Covariance between the random effects was checked. Constant, proportional, and combined residual error models were explored as per the Monolix (2019 R2, Lixoft, Antony, France) library, and the one that gave the smallest Bayesian Information Criteria (BIC) value was retained. Model parameters were estimated by the Stochastic Approximation Expectation‐Maximization (SAEM) algorithm implemented in Monolix.

### 2.3 Survival model

The parametric time‐to‐event (TTE) model was developed to allow covariate evaluation and to enable prediction purposes. The TTE model was selected among exponential, Weibull, Gompertz, log‐logistic, and uniform functions based on a combination of statistical criteria (e.g., BIC), model parsimony, goodness‐of‐fit, and visual prediction check (VPC) plots.

Model parameter estimation was performed using the Monolix SAEM algorithm. The survival model linked tumor response (TGI) and prognostic factors (covariates) to a survival endpoint (e.g., PFS). The covariates included in the analyses are summarized in Table 1. The covariate search step was conducted in Monolix using a stepwise covariate modeling method (SCM), including a set of iterations of forward selection followed by a set of iterations of backward selection, using an inclusion *p*‐value of 0.05 and an exclusion *p*‐value of 0.01.

**TABLE 1:** Baseline disease characteristics and demographics

Covariate	CC‐4047‐MM‐003 (*N* = 455)	CC‐4047‐MM‐007 (*N* = 559)	CC‐5013‐MM‐009 (*N* = 353)
Age, median (min, max)	64.0 (35.0, 87.0)	68.0 (27, 89)	64.0 (36.0, 86.0)
Age distribution, *n* (%)			
≤65	248 (54.5)	243 (43.5)	197 (55.8)
>65	207 (45.5)	316 (56.5)	156 (44.2)
Stratification on age			
≤75	419 (92.1)	466 (83.4)	308 (87.3)
>75	36 (7.9)	93 (16.6)	45 (12.7)
Sex (%)			
Male	268 (58.9)	302 (54.0)	210 (59.5)
Female	187 (41.1)	257 (46.0)	143 (40.5)
Race (%)			
Asian	4 (0.9)	22 (3.9)	7 (2.0)
Black or African American	7 (1.5)	21 (3.8)	42 (11.9)
White	357 (78.5)	471 (84.3)	289 (81.9)
Other	4 (0.9)	39 (7.0)	15 (4.2)
Not collected	83 (18.2)	6 (1.1)	NA

Baseline serum ß2‐microglobulin level (%)^a^, ^b^	**<3.5 mg/L**	136 (29.9)	**<3.5 mg/L**	303 (54.2)	**≤2.5 mg/L**	103 (29.2)
	**3.5 to <5.5 mg/L**	150 (33.0)	**3.5 to <5.5 mg/L**	159 (28.4)	**>2.5 mg/L**	250 (70.8)
	**≥5.5 mg/L**	148 (32.5)	**≥5.5 mg/L**	97 (17.4)		
Number of prior anti‐myeloma therapies, median (min, max)	5.0 (2.0, 17.0)		2.0 (1.0, 5.0)		2.0 (0,8.0)	
Baseline ECOG performance status, *n* (%)						
0	146 (32.1)		286 (51.2)		159 (45.0)	
1	224 (49.2)		240 (42.9)		166 (47.0)	
2	77 (16.9)		33 (5.9)		20 (5.7)	
3	3 (0.7)				0	
Missing	5 (1.1)				8 (2.3)	

Abbreviations: ECOG, Eastern Cooperative Oncology Group; NA, not applicable.

^a^ Baseline serum ß2‐microglobulin levels were stratified based on different criteria per study.

^b^ Twenty‐one patients missing from the CC‐4047‐MM‐003 study.

In addition, a Cox hazard model was developed using the coxph function (R, version 3.6.1) to assess the covariate effect, and Kaplan–Meier curves were plotted.

## 3 RESULTS

### 3.1 Study summary

Three clinical studies (Table 1) with a total of 1367 subjects were included in the analysis. A description of the study is provided in Section 2. Across the three studies, subjects had similar age and sex distributions, with the majority of subjects being white (>75%). Baseline disease characteristics were different. Patients in the CC‐4047‐MM‐003 study were generally sicker, receiving a median of seven prior anti‐myeloma therapeutics compared to the other two studies, in which patients were less pretreated. In line with this, the baseline disease status, including the Eastern Cooperative Oncology Group (ECOG), showed severe MM status in subjects from the CC‐4047‐MM‐003 study compared to the CC‐4047‐MM‐007 and CC‐5013‐MM‐009 studies. This was as expected because the CC‐4047‐MM‐003 study was intended for late line patients, including those who were refractory to Len and BTZ, while the CC‐5013‐MM‐009 and CC‐4047‐MM‐007 studies entailed Len and BTZ as treatment regimens, respectively, and therefore were naive to such regimens [17, 18, 19].

### 3.2 TGI model

**FIGURE 1:**
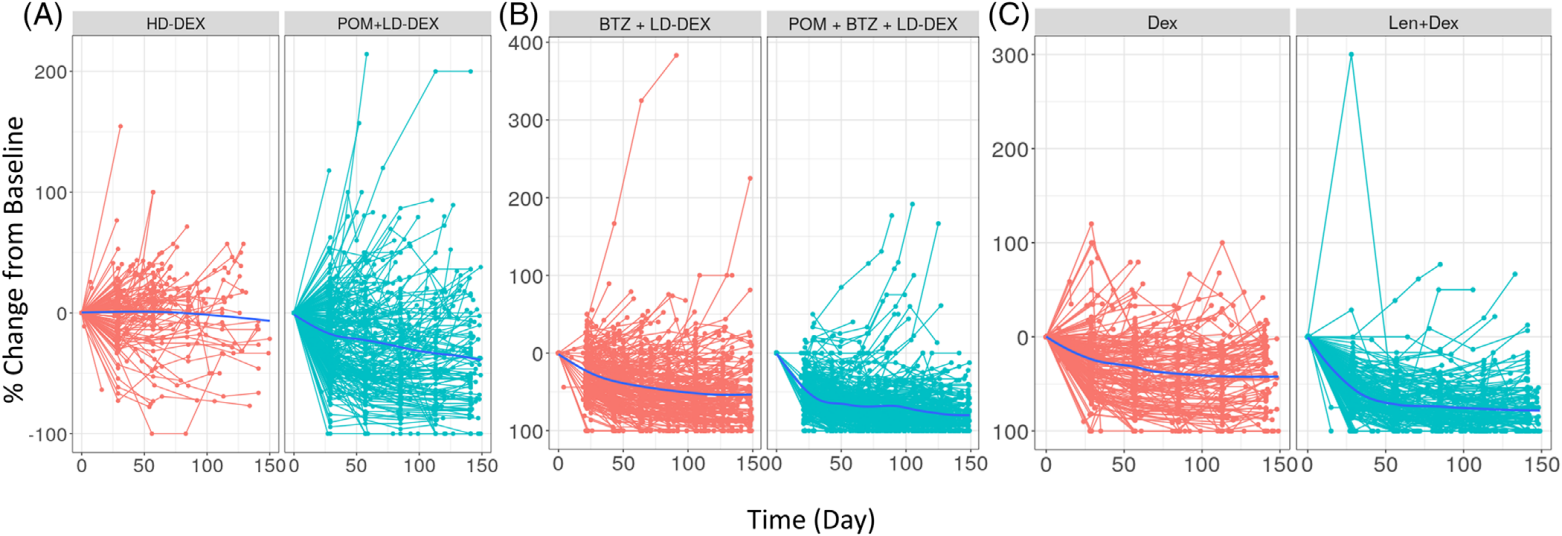
Serum M‐protein kinetic profile from CC‐4047‐MM‐003 (A), CC‐4047‐MM‐007 (B), and CC‐5013‐MM‐009 (C) studies. BTZ + LD‐dex: bortezomib plus low‐dose dexamethasone; dex: dexamethasone; HD‐dex: high‐dose dexamethasone; Len + dex: lenalidomide plus dexamethasone; POM + BTZ + LD‐dex: pomalidomide plus bortezomib plus low‐dose dexamethasone; POM + LD‐dex: pomalidomide plus low‐dose dexamethasone. Solid blue line: locally weighted scatterplot smoothing line

Among 1367 subjects with M‐protein data at any timepoint, 1267 (92.7%) had data that could be used to develop the TGI model for M‐protein. The remaining subjects had missing baseline at the time of the first dose and therefore were excluded from the modeling. The M‐protein percentage change from baseline (PCHG) over time by study and treatment regimen is shown in Figure 1. In each study, the treatment arm consistently showed deeper M‐protein reduction than the control arm, suggesting improvement of efficacy.

**FIGURE 2:**
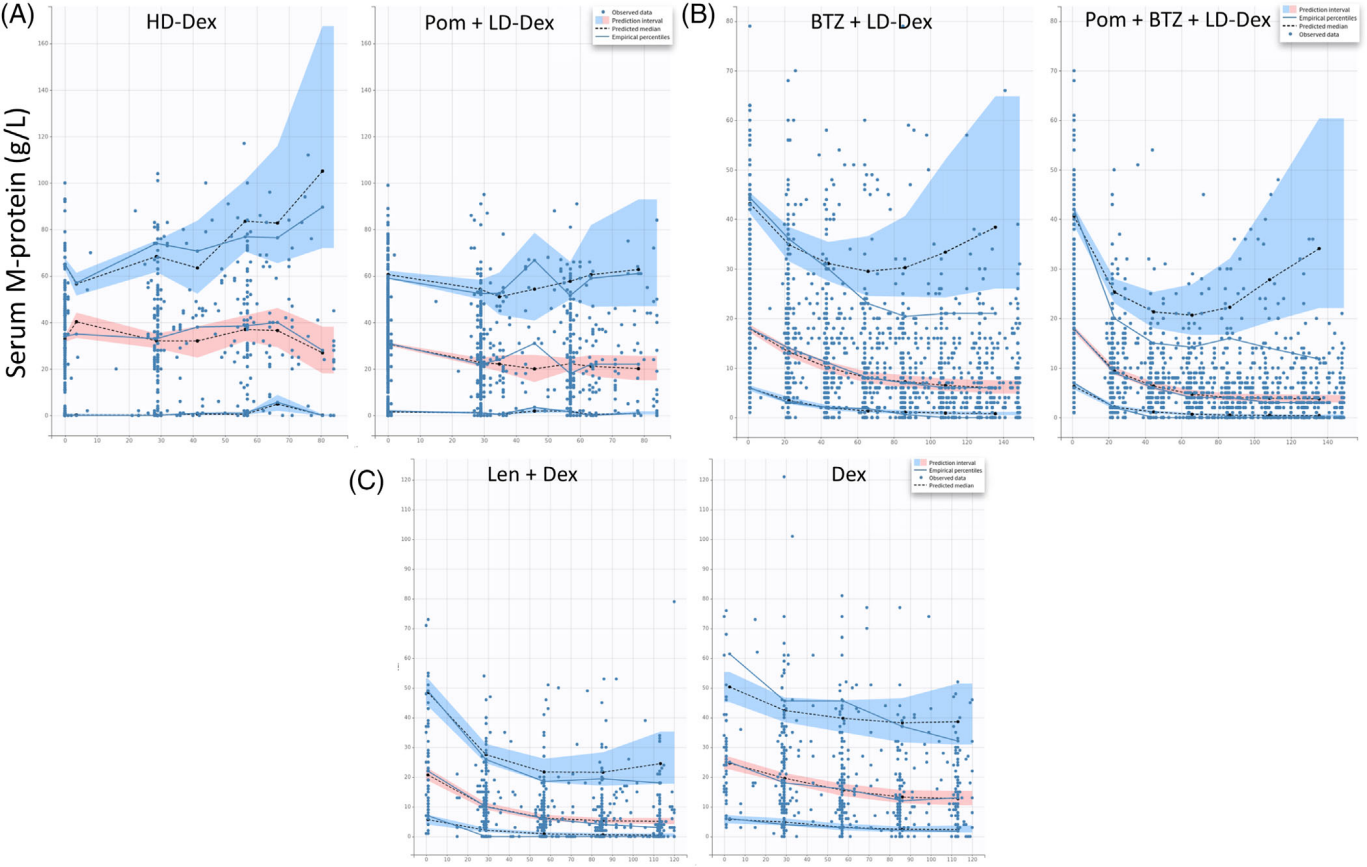
Visual predictive check plots of the tumor growth inhibition (TGI) model. (A) CC‐4047‐MM‐003. (B) CC‐4047‐MM‐007. (C) CC‐5013‐MM‐009. *x*‐axis: time (day); *y*‐axis: serum M‐protein concentration (g/L). The blue dots show the serum M‐protein. The blue lines show the empirical 10th, 50th, and 90th percentiles calculated directly from the population data. The blue‐shaded areas denote the confidence intervals of the 10th and 90th percentiles from the model, and the pink shaded area shows the 50th percentile. The dashed black lines represent the predicted percentiles

The parameters of the TGI model are presented in Table 2. Across three studies, the parameters were estimated with good precision. A visual predictive check (Figure 2) suggested that the model was adequate to describe the data.

**TABLE 2:** Tumor growth inhibition model

	CC‐4047‐MM‐003		CC‐4047‐MM‐007		CC‐5013‐MM‐009	
	Value	RSE (%)	Value	RSE (%)	Value	RSE (%)
Fixed effect						
SR1 (/day)	0.01330	18.7	0.03000	4.3	0.03000	5.0
SR2 (/day)	0.00373	7.7	0.01500	6.5	0.00780	12.6
PR (/day)	0.00383	5.6	0.00095	8.6	0.00093	13.1
Random effect^a^						
Omega (SR1)	1.46	10.3	0.65	5.4	1.2	9.7
Omega (SR2)	1.06	6.4	0.87	6.7	0.6	8.8
Omega (PR)	0.77	5.6	1.57	4.6	1.2	8.6
Error model parameters^b^						
A	0.35	5.6	0.55	3.9	1.2	8.4
B	0.06	3.7	0.10	3.4	0.2	6.8

*Note*: PR, tumor growth rate; SR1, treatment arm tumor inhibition rate (CC‐4047‐MM‐003: Pom + LD‐dex; CC‐4047‐MM‐007: Pom + BTZ + LD‐dex; CC‐5013‐MM‐009: Len + dex); SR2, control arm inhibition rate (CC‐4047‐MM‐003: HD‐dex; CC‐4047‐MM‐007: BTZ + LD‐dex; CC‐5013‐MM‐009: dex).

Abbreviations: BTZ, bortezomib; dex, dexamethasone; HD‐dex, high‐dose dexamethasone; LD‐dex, low‐dose dexamethasone; Len, lenalidomide; Pom, pomalidomide; RSE, relative standard error.

^a^ Log‐normal distribution, that is, log(SR1_individual) = log(SR1_pop) + omega (SR1).

^b^ *y* = *f* + (*A* + *B* × *f*) × *e*, where *f* denotes the structural model and *e* is the base of the natural logarithm.

**FIGURE 3:**
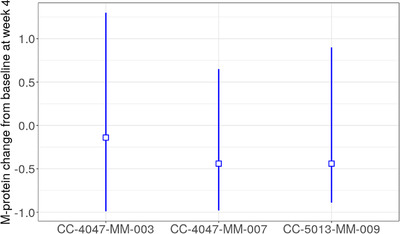
Model‐predicted M‐protein change from baseline at week 4. Blue vertical lines: range of week 4 change from baseline (min to max). Blue squares denote median

Across the studies, the treatment arm TGI rate (SR1) was consistently higher than that from the control arm (SR2), as demonstrated by the two‐ to threefold difference, which was in line with the observation (Figure 1), suggesting the significant improvement of the experimental arm compared to the controls. SR1 differed among studies, with the lowest value in the CC‐4047‐MM‐003 study. Similarly, PR (tumor progression rate) was highest (∼4‐fold higher) in the CC‐4047‐MM‐003 study than in other studies. Of note, similar SR1 and PR were observed between the CC‐4047‐MM‐007 and CC‐5013‐MM‐009 studies, although different treatment regimens were given. This was seen in Figure 3 and Table S1, where the model‐predicted M‐protein change from baseline at week 4 was plotted and summarized for each study, indicating relatively less M‐protein inhibition in the CC‐4047‐MM‐003 study and comparable between the CC‐4047‐MM‐007 and CC‐5013‐MM‐009 studies.

Overall, the TGI model well characterized the M‐protein kinetics among studies.

### 3.3 Survival model

**FIGURE 4:**
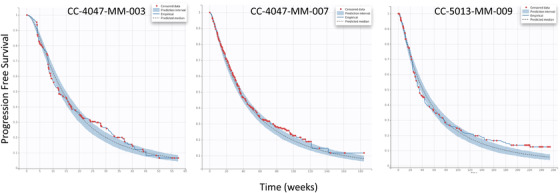
Visual predictive check plots of the progression‐free survival (PFS) final model. *x*‐axis: time (week); *y*‐axis: probability of PFS. Red dots: censored data; blue shaded area: 90% confidence interval from the model; solid blue line: empirical PFS curve; dashed black line: predicted median PFS curve

**FIGURE 5:**
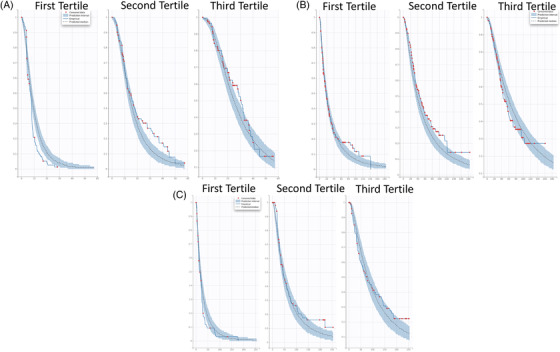
Visual predictive check plots of progression‐free survival (PFS) stratified by M‐protein change from baseline at week 4. (A) CC‐4047‐MM‐003. First tertile: ‐0.01 to 1.28; second tertile: ‐0.26 to ‐0.02; third tertile: ‐0.99 to ‐0.26. (B) CC‐4047‐MM‐007. First tertile: ‐0.32 to 0.65; second tertile: ‐0.54 to ‐0.32; third tertile: ‐0.98 to ‐0.54. (C) CC‐5013‐MM‐009. First tertile: ‐0.28 to 0.90; second tertile: ‐0.55 to ‐0.28; third tertile: ‐0.89 to ‐0.55. *x*‐axis: time (week); *y*‐axis: probability of PFS. Red dots: censored data; blue shaded area: 90% confidence interval from the model; solid blue line: empirical PFS curve; dashed black line: predicted median PFS curve

**FIGURE 6:**
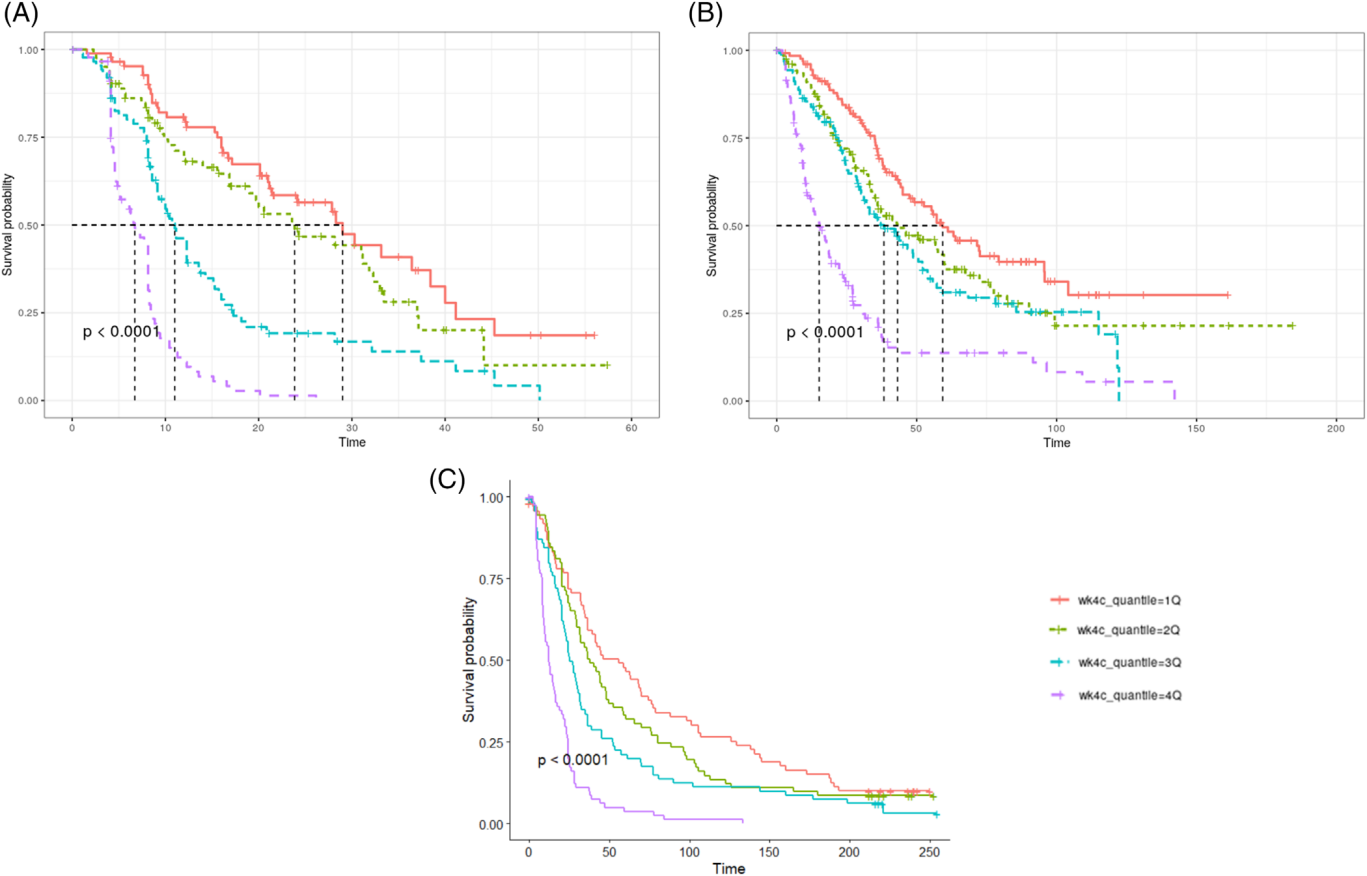
Cox hazard model of progression‐free survival (PFS). *x*‐axis: time (week); *y*‐axis: probability of PFS. The *p*‐value is the log‐rank test comparing the groups. Color of Kaplan–Meier plot: M‐protein change from baseline at week 4 quantiles. (A) CC‐4047‐MM‐003. 1Q: ‐0.99 to ‐0.36; 2Q: ‐0.36 to ‐0.14; 3Q: ‐0.14 to 0.03; 4Q: 0.03 to 1.28. (B) CC‐4047‐MM‐007. 1Q: ‐0.98 to ‐0.60; 2Q: ‐0.60 to ‐0.44; 3Q: ‐0.44 to ‐0.27; 4Q: ‐0.27 to 0.65. (C) CC‐5013‐MM‐009. 1Q: ‐0.89 to ‐0.60; 2Q: ‐0.60 to ‐0.44; 3Q: ‐0.44 to ‐0.21; 4Q: ‐0.21 to 0.90

A parametric model for progression survival (PFS) was developed. The Weibull TTE model was selected as the base model based on BIC, diagnosis plot, and model parsimony. The model parameters were estimated with good precision, as demonstrated by the precision (%relative standard error [RSE]; Table S2) and VPC plots (Figure S1), suggesting adequate model performance. Based on the base model, stepwise covariate searches were performed as stated in Section 2. Notably, beyond the covariates from Table 1, M‐protein reduction from baseline was calculated at the subject level based on the TGI model for weeks 2, 4, 6, 8, 10, and 12. The objective was to identify an early prognostic marker for PFS. Initially, M‐protein changes from baseline at all time‐cuts were tested in the covariates step for the CC‐4047‐MM‐003 study. The SCM algorithm only retained week 4 change in the final model, suggesting that M‐protein change on week 4 exerted the most significant effect on PFS compared to other timepoints, that is, week 8. This finding was then carried over to CC‐4047‐MM‐007 and CC‐5013‐MM‐009 studies, in which only M‐protein week 4 change from baseline plus covariates from Table 1 were tested. Consistently, the M‐protein change parameter was retained in the final model (Table 3), highlighting its strong correlation with PFS across studies. Table 3 summarized the final PFS model with covariate effects. Across the three studies, the models were robust and adequate to describe the PFS data, as demonstrated by the VPC plots (Figure 4). From the final model, a deeper M‐protein change in week 4 was associated with longer PFS, as demonstrated in Figure 5, in which the time to 50% PFS increased from the first tertile (low M‐protein reduction) to the third tertile (high M‐protein reduction). In parallel with this, Kaplan–Meier curves were plotted and stratified by week 4 change quantiles. A Cox hazard model was carried out to assess the week 4 change effect on PFS. Consistently, the results suggested that the M‐protein week 4 change from baseline was significantly (*p* < 0.0001) correlated with PFS across all the studies, with deeper M‐protein inhibition associated with longer PFS (Figure 6).

**TABLE 3:** Parametric progression‐free survival final model

	CC‐4047‐MM‐003^a^		CC‐4047‐MM‐007^b^		CC‐5013‐MM‐009^c^	
	Value	RSE (%)	Value	RSE (%)	Value	RSE (%)
Fixed effect						
Te	12.98	9.3	31.95	13.3	28.03	16.1
P	8.45	17.5	9.30	14.7	7.31	13.1
M‐protein week 4 change on Te	−1.92	8.6	−2.05	9.0	−0.03	8.3
Baseline serum ß2‐microglobulin^d^ level: >2.5 mg/L on Te	NA	NA	NA	NA	−0.53	24.0
Baseline serum ß2‐microglobulin level: ≥5.5 mg/L on Te	−0.13	79.2	−0.90	14.7	NA	NA
Baseline serum ß2‐microglobulin level: ≥3.5 and <5.5 mg/L on Te	NA	NA	−0.42	27.2	NA	NA
Baseline serum ß2‐microglobulin level: <3.5 mg/L on Te	0.21	49.8	NA	NA	NA	NA
ECOG^e^ = 0 on Te	−0.24	39.3	−0.31	32.2	NA	NA
Prior anti‐myeloma regimen^f^ = 1	NA	NA	−0.32	32.4	−0.30	40.8
Random effect						
Omega (Te)	0.64	6.8	0.94	4.8	1.2	5.2
Omega (p)	1.14	11.4	0.98	10.1	0.8	15.5

Abbreviations: NA, not applicable; RSE, relative standard error.

^a^ Log(Te_individual) = log(Te_pop) + (M‐protein week 4 change on Te) × (M‐protein week 4 change) + (baseline serum ß2‐microglobulin level: ≥5.5 mg/L on Te) × (if baseline serum ß2‐microglobulin level: ≥5.5 mg) + (baseline serum ß2‐microglobulin level: <3.5 mg/L on Te) × (if baseline serum ß2‐microglobulin level: <3.5 mg) + (ECOG = 0 on Te) × (if ECOG = 0) + omega (Te).

^b^ Log(Te_individual) = log(Te_pop) + (M‐protein week 4 change on Te) × (M‐protein week 4 change) + (baseline serum ß2‐microglobulin level: ≥5.5 mg/L on Te) × (if baseline serum ß2‐microglobulin level: ≥5.5 mg) + (baseline serum ß2‐microglobulin level: ≥3.5 and <5.5 mg/L on Te) × (if baseline serum ß2‐microglobulin level: ≥3.5 and <5.5 mg/L) + (ECOG = 0 on Te) × (if ECOG = 0) + (prior anti‐myeloma regimen = 1 on Te) × (if prior anti‐myeloma regimen = 1) + omega (Te).

^c^ Log(Te_individual) = log(Te_pop) + (M‐protein week 4 change on Te) × (M‐protein week 4 change) + (baseline serum ß2‐microglobulin level: >2.5 mg/L on Te) × (if baseline serum ß2‐microglobulin level: >2.5 mg) + (prior anti‐myeloma regimen = 1 on Te) × (if prior anti‐myeloma regimen = 1) + omega (Te).

^d^ Reference: baseline serum ß2‐microglobulin level: ≤2.5.

^e^ Reference: ECOG > 0.

^f^ Reference: prior anti‐myeloma regimen > 1.

**FIGURE 7:**
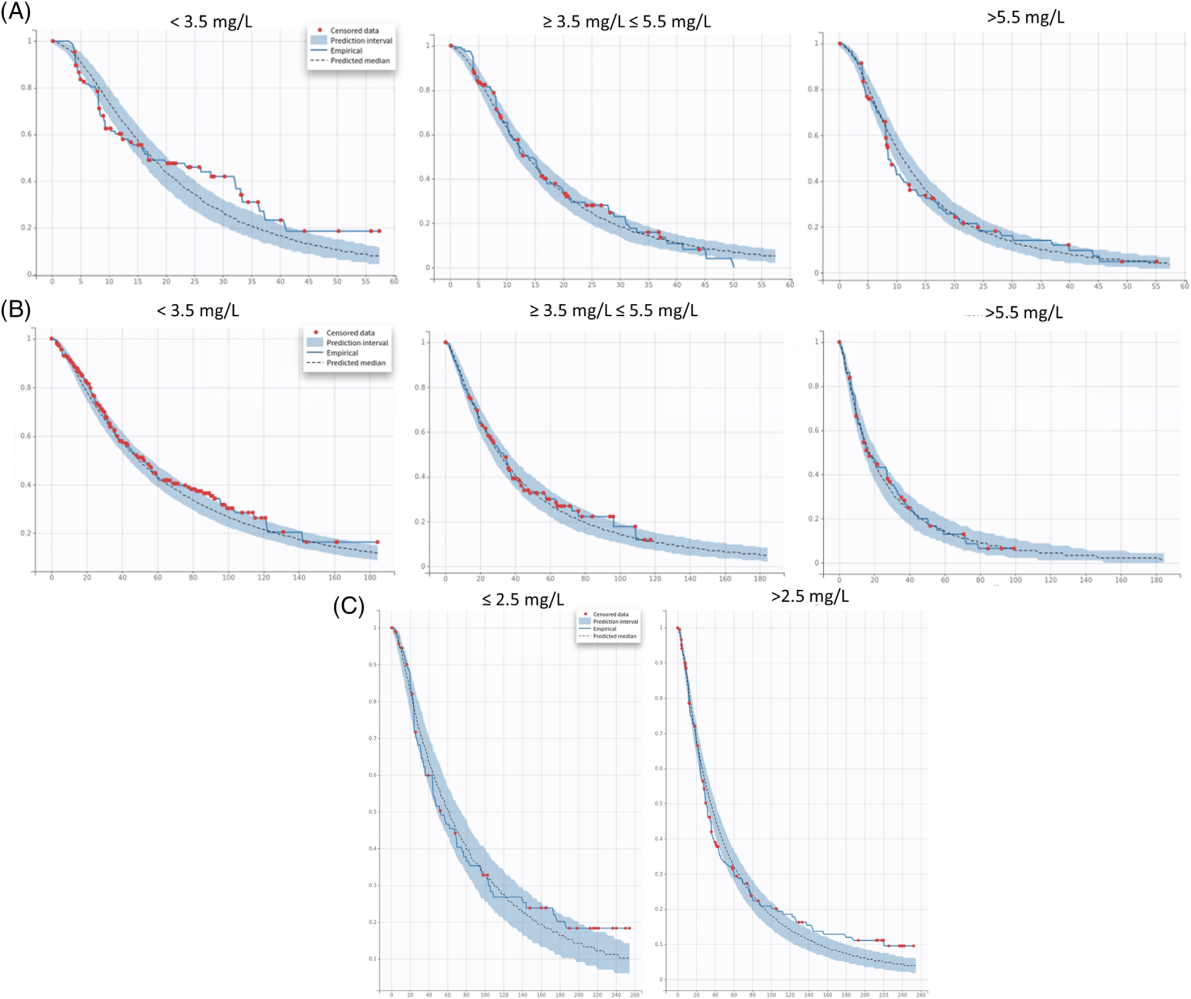
Visual predictive check plots of progression‐free survival (PFS) stratified by baseline serum ß2‐microglobulin level. (A) CC‐4047‐MM‐003. (B) CC‐4047‐MM‐007. (C) CC‐5013‐MM‐009. *x*‐axis: time (week); *y*‐axis: probability of PFS. Red dots: censored data; blue shaded area: 90% confidence interval from the model; solid blue line: empirical PFS curve; dashed black line: predicted median PFS curve

**FIGURE 8:**
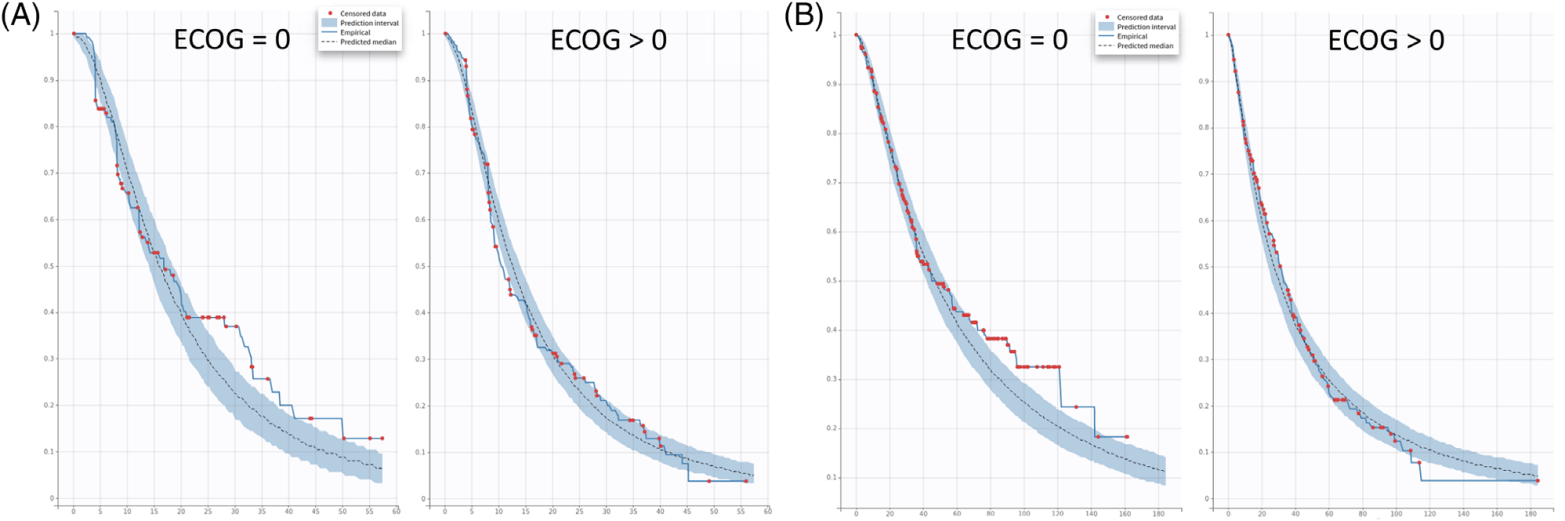
Visual predictive check plots of progression‐free survival (PFS) stratified by Eastern Cooperative Oncology Group (ECOG). (A) CC‐4047‐MM‐003. (B) CC‐4047‐MM‐007. *x*‐axis: time (week); *y*‐axis: probability of PFS. Red dots: censored data; blue shaded area: 90% confidence interval from the model; solid blue line: empirical PFS curve; dashed black line: predicted median PFS curve

**FIGURE 9:**
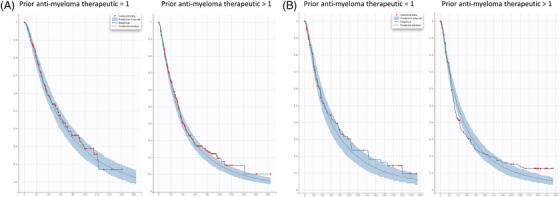
Visual predictive check plots of progression‐free survival (PFS) stratified by prior anti‐myeloma therapeutics. (A) CC‐4047‐MM‐007. (B) CC‐5013‐MM‐009. *x*‐axis: time (week); *y*‐axis: probability of PFS. Red dots: censored data; blue shaded area: 90% confidence interval from the model; solid blue line: empirical PFS curve; dashed black line: predicted median PFS curve

Additionally, the baseline serum ß2‐microglobulin level was included in the final models for all three studies. Due to the different stratification criteria between studies, baseline serum ß2‐microglobulin levels were grouped into either >2.5 mg/L versus ≤2.5 mg/L (CC‐5013‐MM‐009) or <3.5 mg/L versus ≥3.5 mg/L and <5.5 mg/L versus ≥5.5 mg/L (CC‐4047‐MM‐003 and CC‐4047‐MM‐007) categories. Regardless of this, an association between lower ß2‐microglobulin levels and longer PFS was observed for all studies (Table 3 and Figure 7). ECOG (ECOG = 0 vs. ECOG > 0) was incorporated into the final models of CC‐4047‐MM‐003 and CC‐4047‐MM‐007 studies, and a prior anti‐myeloma regimen (prior anti‐myeloma regimen = 1 vs. >1) was retained in the final model of CC‐4047‐MM‐007 and CC‐5013‐MM‐009 studies. Consistently, a less severe (ECOG = 0 compared with >0) and less prior anti‐myeloma regimen (prior regimen = 1 compared with >1) were associated with longer PFS (Table 3 and Figures 8 and 9).

## 4 DISCUSSION

Leveraging early biomarker data to prognose long‐term survival has been used to support the clinical development of multiple oncology programs. The concept of TGI‐survival analysis, compared with the widely accepted response criteria (e.g., RECIST), has demonstrated superiority by taking holistic longitudinal tumor size data into consideration to prognose the long‐term clinical benefits [21]. In fact, Feng et al. [7] suggested that the continuous measure of the tumor response (tumor size reduction) might be a more clinically relevant prognostic factor of OS than overall response rate (ORR) by RECIST criteria, based upon data from a phase III study of ipilimumab.

Similar to other oncology programs, in the space of MM, the early prognosis of drug efficacy signals for a novel treatment regimen is thought to streamline clinical drug development. The TGI‐survival analysis framework is one of the good options to meet this need. However, unlike direct tumor size measurement in solid tumor settings, hematology malignancies, including MM, require an additional step to tease out reliable biomarkers as tumor burden surrogates, which inevitably poses challenges. Given the biological plausibility [1], serum M‐protein appeared appropriate for TGI‐survival analysis in MM. This argument can be further validated by several recent meta‐analyses based on clinical studies, which unequivocally highlighted the strong prognostic capability of M‐protein reduction for long‐term survival in MM patients [11, 12, 13].

Herein, we systemically examined the application of TGI‐survival analysis in MM by using data from three phase III clinical studies covering different treatment regimens and patient populations. We developed TGI models for each study and subsequently linked the TGI with PFS. The data clearly demonstrated that early M‐protein changes (i.e., week 4 change from baseline) were significantly correlated with PFS.

In the TGI model, the tumor inhibition rate (SR) was separately estimated for the experimental arm (SR1) and the control arm (SR2). This was informed by the findings from clinical studies, which showed that there was a significantly better clinical efficacy response in the experimental arms than in the control arms (efficacy endpoints: OS for CC‐5013‐MM‐009; PFS for CC‐4047‐MM‐003 and CC‐4047‐MM‐007) [17, 18, 19]. In line with these findings, the SR from experimental arms was consistently higher than controls, indicating deeper inhibition of M‐protein levels.

Of note, the patient disease characteristics (i.e., baseline tumor burden and treatment lines) also influenced M‐protein changes. Across the three studies, the CC‐4047‐MM‐003 study had a high tumor growth parameter (PR) and low inhibition (SR) (Table 2), which suggested that the CC‐4047‐MM‐003 study may have the lowest treatment effect (Figure 3). This was consistent with the study results. For instance, the median PFS in the CC‐4047‐MM‐003 study was 4.0 months, and in the CC‐4047‐MM‐007 study, it was 11.2 months, providing a comparable median follow‐up time (10.0 months vs. 15.9 months) [17, 18]. Such an observation can be partly explained by the apparent patient population difference, that is, the heavily pretreated patients from the CC‐4047‐MM‐003 study, compared to the relatively earlier treatment lines of patients from the other two studies, may cause huge complexities to knock‐down the M‐protein.

In addition, the similar tumor inhibition (SR1: experiment arm) and growth rate (PR) (Table 2) and M‐protein inhibition effect at week 4 (Figure 3) between CC‐4047‐MM‐007 and CC‐5013‐MM‐009 studies (both in relative early lines), although varied in terms of treatment regimens, further validated the argument that patient disease characteristics (i.e., baseline tumor burden and treatment lines) were remarkable determinants of M‐protein change. It is worth noting that the control arm tumor inhibition effect (SR2) in the CC‐4047‐MM‐007 study (regimen: BTZ + LD‐dex) was approximately twofold that of the CC‐5013‐MM‐009 study (regimen: dex). Given the relatively similar tumor growth rate between the two studies, this result indicated that the BTZ + LD‐dex regimen (from the CC‐4047‐MM‐007 study) may exert a deeper M‐protein inhibition effect than dex (CC‐5013‐MM‐009).

The M‐protein change from baseline parameters was calculated based on the TGI model. Initially, a variety of time‐cuts were used and tested in the model for the CC‐4047‐MM‐003 study as a starting point, ranging from weeks 2 to 12. Univariate analysis (Cox regression) suggested that all the parameters showed statistical significance (*p* < 0.0001). Subsequently, through covariate search (SCM in Monolix), only week 4 was retained in the final parametric PFS model, highlighting the strongest correlation. This was consistent with another publication by Jonsson et al. [11], which utilized data from single‐agent carfilzomib studies to develop TGI‐OS analysis and identified that early change in tumor size (M‐protein) at week 4 was a significant independent predictor for OS. Although other publications used an even longer time‐cut (e.g., week 8) [3, 7], our analysis clearly showed the superiority of week 4 data. In addition, the week 4 time point was suitable for the clinical assessment schedule, especially for clinical studies with outpatient settings, given that many clinical programs (e.g., Len, Pom) administered the investigational drugs on a 28‐day (∼4 weeks) treatment cycle schedule.

Aside from M‐protein changes, baseline serum ß2‐microglobulin, ECOG, and prior anti‐myeloma therapeutics were retained in the final models. Serum ß2‐microglobulin was a key variable of the International Staging System for MM [22] and therefore was considered an indicator of baseline disease burden. Collectively, the results suggested that fewer sick patients (i.e., lower baseline serum ß2‐microglobulin, smaller ECOG score, and earlier therapeutic lines) were associated with longer PFS. This was in line with other publications for MM and solid tumors, which included ECOG and other baseline disease factors in the survival model [3, 11].

Of note, MM subjects come from a heterogeneous group, and the disease can be further classified by the light and heavy chains from the paraproteins produced (i.e., IgA, IgG, and other types). Given that each subtype of paraprotein has different half‐lives [23], for example, the most common IgG subtype has a long half‐life (total IgG half‐life 25.8 days, although there are several subtypes of IgG with different half‐lives [24]), whereas other subtypes, such as IgA (4–6 days [25]) or IgE (2–3 days [26]), have shorter half‐lives, we believe it is relevant and meaningful to assess the impact of disease heterogeneity on the TGI‐PFS model, which takes into account the kinetic profiles of M‐proteins, although several recent similar works did not address this [11, 12]. In the current exercise, we explored the M‐protein types as covariates in the PFS model. Our analyses suggested that there was no significant difference in model performance between the disease subtypes of IgG and IgA, suggesting that the current model can adequately characterize the heterogeneous patient population.

Given the differences in treatment regimens among studies (e.g., Pom + LD‐dex, Len + dex, etc.), the current analyses did not include drug exposure in the model. It is worth noting that based on published population pharmacokinetics (PK) analyses [27, 28], the PK variabilities (e.g., between‐subject variabilities of clearance) were moderate (36%–41%) for Pom and Len. Thus, adding PK exposure for each individual study was not anticipated to substantially alter the results. However, as future efforts, an integrated PK‐TGI‐survival framework can be considered to account for the between‐subject differences and/or dose effect, which can be used to inform dose selection in early dose escalation studies. Several publications have provided contexts for this approach [29, 30]. Moreover, the analysis herein only focused on serum M‐protein as a tumor burden surrogate. Other clinically relevant MM hallmarks [1], including serum‐free light chain (a biomarker with a relatively shorter half‐life) and urine M‐protein, may afford additional resolution for the data interpretation by the model. Notably, the TGI and survival model were developed sequentially in the current exercise, which was in line with other reports [3, 7]. The influences of various M‐protein reduction time‐cuts (e.g., 4 weeks) on PFS were unknown prior to the analyses such that M‐protein simulation needs to be conducted to explore the correlation first. However, as a future effort, a joint model may be developed. Last but not least, the current analyses, although incorporating Pom and Len, which are widely considered standard of care in their respective treatment lines, were restricted to immunomodulatory agents. Given that the therapeutic landscape of MM is evolving rapidly, a meta‐analysis including monoclonal antibodies, antibody–drug conjugates, and cell therapy in addition to small molecule agents will be highly desirable. However, despite the aforementioned limitation, the recent breakthroughs of several novel CelMod programs, including iberdomide [31] and CC‐92480 [32], which have shown promising clinical outcomes, are expected to benefit from this analysis.

In conclusion, the current work systemically examined the application of TGI‐survival analyses in the context of MM. Three phase III studies were used to develop the TGI‐PFS model. Serum M‐protein was used as a tumor burden surrogate. We found that the M‐protein change from week 4 was significantly correlated with PFS. Patient disease characteristics (i.e., baseline tumor burden and treatment lines) were important determinants for tumor inhibition and PFS. This work, instead of helping physicians make treatment decisions in the clinic, aimed to provide insights for MM clinical drug development, including triaging lead compounds, rationalizing early clinical study design, and informing dosing decisions.

## CONFLICTS OF INTEREST

Yiming Cheng, Kevin Hong, Nianhang Chen, Xin Yu, Teresa Peluso, Simon Zhou, and Yan Li are employees and hold equity ownership in Bristol Myers Squibb.

## AUTHOR CONTRIBUTIONS

Yiming Cheng, Kevin Hong, Nianhang Chen, Xin Yu, Teresa Peluso, and Yan Li designed and performed the research. Yiming Cheng and Yan Li contributed to the analysis. Yiming Cheng, Simon Zhou, and Yan Li wrote the paper. All authors made substantial contributions to the conception and design, acquisition of data, or analysis and interpretation of data; took part in drafting the article or revising it critically for important intellectual content; agreed to submit to the current journal; gave final approval of the version to be published; and agreed to be accountable for all aspects of the work. All the authors have reviewed and concurred with the manuscript.

## PATIENT CONSENT STATEMENT

All subjects gave written informed consent prior to enrollment.

## ETHICS STATEMENT

The studies were conducted in accordance with the ethical principles of Good Clinical Practice. All subjects gave written informed consent prior to enrollment. The studies were approved by the Independent Ethics Committee/Institutional Review Boards of the participating center and were conducted according to the Declaration of Helsinki and the ICH Guidelines for Good Clinical Practice.

## Supporting information

Supplementary Figure S1. Visual predictive check plots of the progression‐free survival (PFS) base model
Supplementary Table S2: Parametric progression‐free survival base model

## Data Availability

The datasets are available from the corresponding author upon request.
