# Supplementary material for: Aiding early clinical drug development by elucidation of the relationship between tumor growth inhibition and survival in relapsed/refractory multiple myeloma patients
Source: EJHaem. 2022 Jun 2;3(3):815–27. doi: 10.1002/jha2.494 (PMC9422038; doi:10.1002/jha2.494)
Supplement: Supplementary file 1 — Supplementary Figure S1. Visual predictive check plots of the progression‐free survival (PFS) base model Supplementary Table S2: Parametric progression‐free survival base model [file JHA2-3-815-s001.docx]

**Supplementary Figure S1.** Visual predictive check plots of progression free survival (PFS) base model.


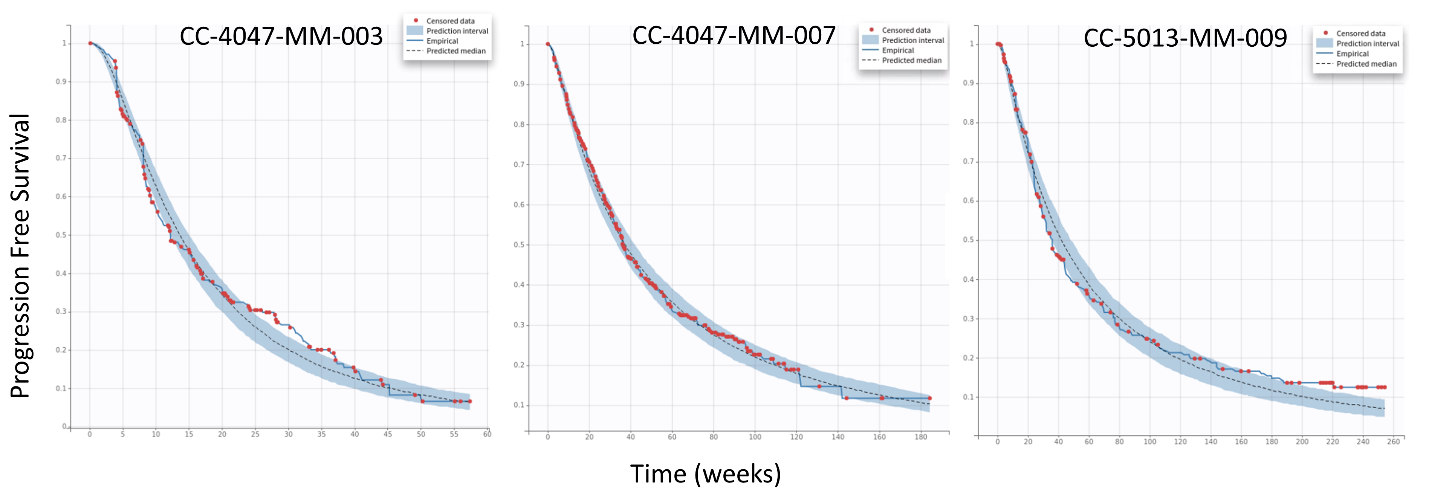
x-axis: time (week); y-axis: probability of PFS. Red dots: censored data; blue shaded area: 90% confidence interval from the model; solid blue line: empirical PFS curve; dashed black line: predicted median PFS curve.

**Supplementary Table S1: Model Predicted Serum M-protein Change from Baseline Statistics**

|  | **CC-4047-MM-003** | **CC-4047-MM-007** | **CC-5013-MM-009** |
| --- | --- | --- | --- |
| 1st Quantile | -0.99 to -0.36 | -0.98 to -0.60 | -0.89 to -0.60 |
| 2nd Quantile | -0.36 to -0.14 | -0.60 to -0.44 | -0.60 to -0.44 |
| 3rd Quantile | -0.14 to 0.03 | -0.44 to -0.27 | -0.44 to -0.21 |
| 4th Quantile | 0.03 to 1.28 | -0.27 to 0.65 | -0.21 to 0.90 |

**Supplementary Table S2: Parametric Progression Free Survival Base Model**

|  | **CC-4047-MM-003** | | **CC-4047-MM-007** | | **CC-5013-MM-009** | |
| --- | --- | --- | --- | --- | --- | --- |
|  | Value | RSE(%) | Value | RSE(%) | Value | RSE(%) |
| **Fixed Effect** | | | | | | |
| Te | 15.7 | 5.7 | 43.23 | 6.5 | 46.87 | 7.9 |
| p | 4.6 | 15.7 | 4.92 | 11.8 | 7.69 | 16.5 |
| **^a^Random Effect** | | | | | | |
| Omega (Te) | 0.9 | 5.4 | 1.23 | 4.8 | 1.2 | 5.2 |
| Omega (p) | 0.3 | 36.8 | 0.56 | 13.7 | 0.8 | 15.5 |

RSE: relative standard error

Te denoted the scale parameter and p represented the shape parameter.

^a^ Log-normal distribution, i.e., log (Te_individual) = log (Te_pop) + omega (Te).
